# Transferrin knockout reveals a tolerance phenotype against *Piscirickettsia salmonis* in Atlantic salmon phagocytes

**DOI:** 10.1186/s13567-025-01607-8

**Published:** 2025-09-25

**Authors:** Madelaine Mejías, Alejandro Sáez, Amanda Escorza, Felipe Galdames-Contreras, Yehwa Jin, Phillip Dettleff, Ingrid Ojeda, Rodrigo Pulgar, Sebastián Escobar-Aguirre

**Affiliations:** 1 Laboratorio de Biología Molecular Marina, Departamento Ciencias Animales, Facultad de Agronomía y Sistemas Naturales, Pontificia Universidad Católica de Chile, Santiago, Chile; 2 Laboratorio de Genómica y Genética de Interacciones Biológicas (LG2IB), Instituto de Nutrición y Tecnología de los Alimento, Universidad de Chile, Santiago, Chile; 3 Center for Research and Innovation in Aquaculture (CRIA), Universidad de Chile, Santiago, Chile; 4 Center for Aquaculture Technologies, San Diego, CA 92121 USA; 5 Escuela de Medicina Veterinaria, Facultad de Agronomía y Sistemas Naturales, Facultad de Ciencias Biológicas y Facultad de Medicina, Pontificia Universidad Católica de Chile, Santiago, Chile; 6 Cermaq Chile S.A. Diego Portales, 2000 - Puerto Montt Los Lagos, Chile

**Keywords:** Vaccines, *Salmo salar*, transferrin, gene editing, *Piscirickettsia salmonis*, SRS

## Abstract

Salmonid rickettsial septicemia (SRS), caused by *Piscirickettsia salmonis*, is a major challenge in Chilean aquaculture. We evaluated the impact of the vaccine on fish survival, bacterial load, and iron metabolism-related gene expression under field conditions. Atlantic salmon received either a pentavalent inactivated vaccine plus a live attenuated *P. salmonis* vaccine (SIA) or a tetravalent control vaccine (SS). While survival was similar early (≤ 28 weeks post-seawater transfer), SIA-vaccinated fish showed greater survival by week 41 (85% vs. 52%). Quantitative PCR confirmed a reduced bacterial load in the SIA group during active infection. Expression analysis revealed distinct temporal regulation of iron metabolism genes: ferritin and hepcidin were upregulated in freshwater, whereas transferrin and its receptor were elevated in seawater in the SIA group, suggesting a link between iron homeostasis and vaccine-induced protection. To further investigate the role of transferrin, we generated transferrin-knockout (TF-KO) phagocytes using CRISPR/Cas9. In vitro infection assays revealed that, compared with wild-type (TF-WT) cells, TF-KO cells presented reduced cytopathic effects, decreased formation of *P. salmonis*-containing vacuoles (PCVs), and improved viability. Surprisingly, no differences in bacterial load or iron-related gene expression were detected between TF-KO and TF-WT cells, indicating that transferrin disruption did not directly alter iron homeostasis. Global transcriptomic analysis revealed 311 differentially expressed genes in TF-KO cells, with functional enrichment in metal-binding and zinc-dependent processes but no direct association with iron metabolism. These findings suggest that transferrin deficiency confers an infection-tolerant phenotype through transcriptional reprogramming unrelated to iron regulation, highlighting novel mechanisms of host‒pathogen interactions and potential avenues for SRS control in aquaculture.

**Supplementary Information:**

The online version contains supplementary material available at 10.1186/s13567-025-01607-8.

## Introduction

Salmonid rickettsial septicaemia (SRS), caused by the intracellular bacterium *Piscirickettsia salmonis*, is a significant infectious disease affecting farmed salmonids, leading to substantial economic losses in the Chilean aquaculture industry [1]. Vaccination is widely used as a preventive strategy to mitigate the impact of SRS. However, information on the immune response and protection conferred by these vaccines under field conditions remains limited, and the consensus suggests that their long-term efficacy is suboptimal [2, 3]. Hence, one of the most extensively studied aspects in recent years for improving SRS vaccines is the host‒pathogen interaction between *P. salmonis* and salmonids, identifying iron metabolism as a crucial factor influencing fish tolerance to infection. These findings agree with previous studies reporting that *P. salmonis* is a ferrophilic bacterium with pronounced tropism for the head kidney, the primary hematopoietic organ in salmon [4]. In response, the iron availability of infected salmonids is limited to restrict bacterial replication in this tissue by modulating the expression of genes encoding iron transporters (transferrin, transferrin receptor, and ferroportin), iron storage proteins (ferritins), and iron metabolism regulatory proteins (hepcidin) [5]. This knowledge has contributed to the development of novel control strategies, including dietary supplementation with the iron chelator deferiprone [6] and vaccines based on chimeric iron transport protein antigens [7], both of which have significantly reduced fish mortality following *P. salmonis* exposure.

Although the evidence in fish is still limited, some studies in mammals indicate that iron homeostasis not only influences innate immunity but also plays a crucial role in adaptive immunity, which is fundamental to vaccine efficacy [8]. For example, T-cell proliferation, as well as T-cell activation and thymic cellularity, are impaired in the absence of iron, as their activation is coordinated through iron uptake via the transferrin receptor (TfR1) via an IL-2-dependent pathway [9]. Furthermore, some iron nanoparticle-based adjuvants have been shown to enhance Th1, Th17, and CD8 + T-cell immune responses, mainly in the lungs of mice, emphasizing the role of iron in adaptive immunity [10]. In humans, individuals with serum iron deficiency exhibit significantly lower antibody responses than those with normal iron levels do, whereas increased iron availability has been linked to increased B-cell proliferation [11–14]. In Atlantic salmon (*Salmo salar*), Martinez and coworkers recently reported that plasma iron levels decreased while hepatic iron levels increased following vaccination with both live and inactivated *P. salmonis*. Additionally, the transcript abundance of key proteins involved in cellular iron metabolism differs between vaccinated and nonvaccinated fish [15], suggesting that the relationships among iron metabolism, vaccine efficacy, and infectious disease control may be governed by conserved mechanisms across vertebrates.

Given the background described above, this study aimed to examine the effects of vaccination *against P. salmonis* in Atlantic salmon, with a particular focus on the transcriptional abundance of key iron metabolism-related genes in the head kidney. To achieve this goal, fish were immunized with a multivalent vaccine containing antigens from *P. salmonis* (SIA vaccine), whereas a sham vaccine (SS vaccine) lacking *P. salmonis* antigens was used as a control to account for potential cross-reactive effects from antigens targeting other pathogens. On the basis of these findings, we employed CRISPR/Cas9-mediated gene editing to knock out transferrin genes in phagocytes derived from the Atlantic salmon head kidney (SHK-1 cell line) to assess the impact of transferrin deficiency on the phagocytic response to *P. salmonis* infection in vitro. Finally, we conducted transcriptomic profiling (RNA-Seq) of wild-type and gene-edited cell lines to identify differentially expressed genes and biological processes potentially involved in the differential cellular response to *P. salmonis* infection.

## Materials and methods

### Fish farmed under field conditions, vaccination and sampling

**Figure 1:**
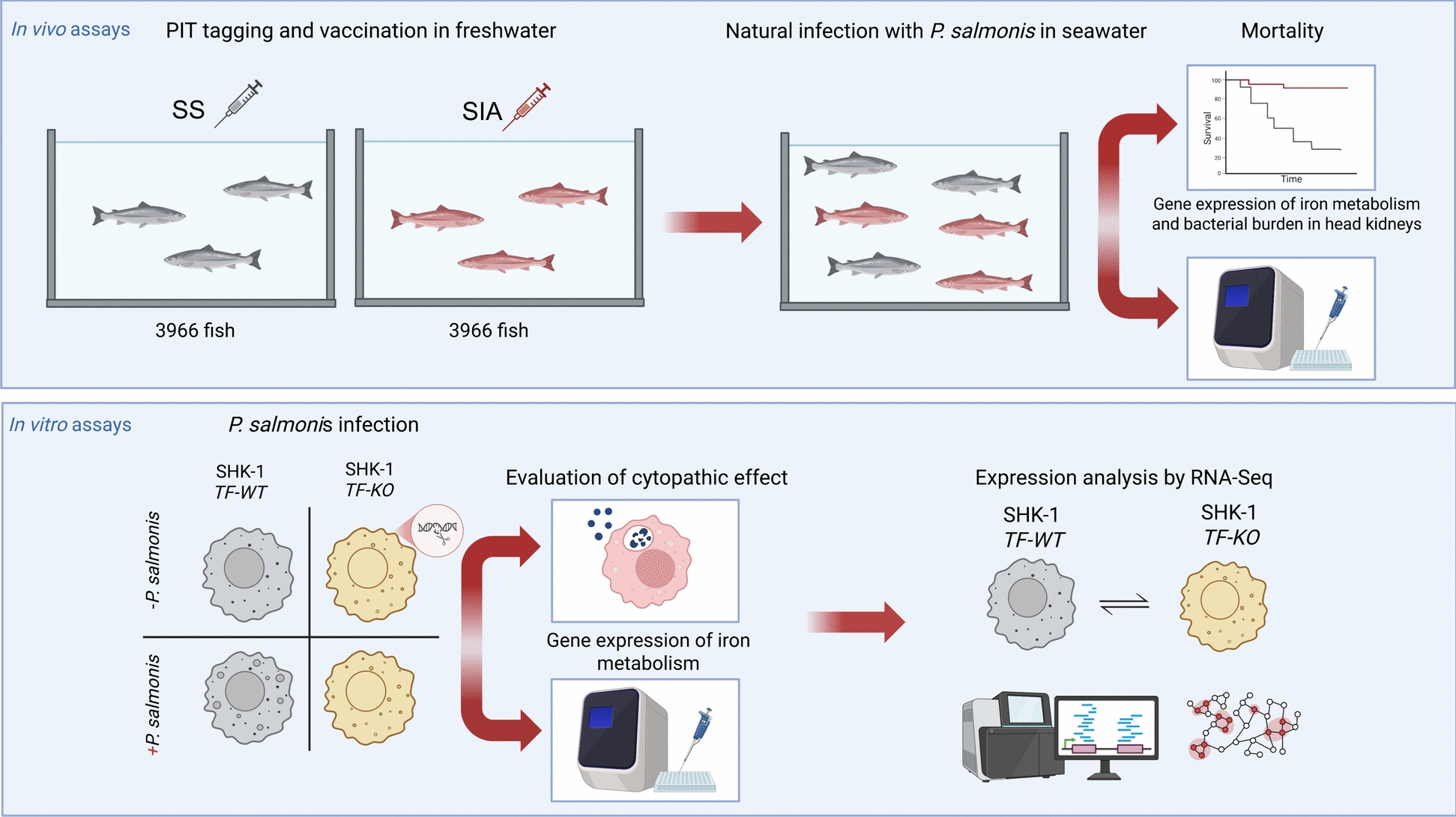
**Scheme of the experimental design.** Overview of the in vivo and in vitro assays used to evaluate the role of transferrin (TF) in the response to *Piscirickettsia salmonis*. Upper panel: Atlantic salmon (*Salmo salar*) smolts were PIT-tagged and vaccinated with a commercial pentavalent inactivated vaccine mixed with a monovalent live attenuated vaccine against *P. salmonis* (SRS inactivated + attenuated, SIA group). Another group was also subjected to an intraperitoneal injection of a tetravalent attenuated vaccine containing no antigens against *P. salmonis* (SRS sham reference, SS group). Mortality rates, the expression of iron metabolism-related genes, and the bacterial load in head kidneys were assessed. Lower panel: SHK-1 cell lines, either wild-type (TF-WT) or with transferrin knocked out (TF-KO), were infected with *P. salmonis* in vitro. Cytopathic effects and iron-related gene expression were analysed between conditions.

Presmolts of Atlantic salmon (80 to 100*g*) were obtained from the Rio Pescado hatchery (SIEP code 100,323) Puerto Varas, Chile. Twenty days before vaccination, the health of the fish was assessed, and PIT tags were inserted into the abdominal cavity of the fish via injection. Only healthy fish with active PIT tags were vaccinated in freshwater for subsequent trials. To evaluate the effect of vaccination on fish mortality due to SRS under commercial farming conditions, a group of 3966 fish were intraperitoneally vaccinated with a commercial pentavalent inactivated vaccine mixed with a monovalent live attenuated vaccine against *P. salmonis* (SRS inactivated + attenuated, SIA group). Another group of 3966 fish was also subjected to an intraperitoneal injection of a tetravalent attenuated vaccine containing no antigens against *P. salmonis* (SRS sham reference, SS group). The commercial vaccines used in this study were selected according to provisional registration by the Agricultural and Livestock Service of Chile (SAG) [SAG, 2024], and their formulation details are shown in Table 1. Both fish groups (SIA and SS) were subsequently transferred to seawater (Colaco experimental seawater centre in Puerto Montt, Los Lagos region Chile) and remained in the same sea pen throughout the experiment. The mortality of both groups of fish (pit-tagged fish) due to the SRS outbreak was recorded weekly. All mortalities were reported to SERNAPESCA, and its confirmation was performed either by indirect immunofluorescence or by necropsy with signs congruent with SRS and confirmed by an ETECMA external laboratory: CCS-MIM-182–551, CCS-RSN-218–112, and CCS-JS-201–069-1. The sampling times used in this study (T1, T2, T3, T4, T5 and T6) are listed and described in Table 2. Five live fishes were sampled from each condition group (SIA and SS) from T1 to T6. To measure the bacterial load of *P. salmonis* and the relative abundance of transcripts linked to iron metabolism in Atlantic salmon, the head kidney was collected and placed into a 2 mL microtube with RNAlater (Sigma‒Aldrich, USA) stored at -80 °C until further analysis. A scheme of the experimental design is shown in Figure 1.

**Table 1:** **Formulation of the vaccines used in this study**

Treatment name	Formulation
SRS sham (SS)	Inactivated vaccine against: IPNv, ISAv, *Aeromonas salmonicida*, *Vibrio ordalii*
SRS Inactivated + attenuate (SIA)	Inactivated vaccine against: IPNv, ISAv, *Aeromonas salmonicida*, *Vibrio ordalii*, Inactivated + Live Attenuated vaccine of *P. salmonis*

**Table 2:** **Sampling times, accumulated thermal units (ATUs) and water stage**

Time	ATUs post- vaccination	ATUs post- seawater-exposure	Week	Water Stage
T1	452 ± 18	–	12	Fresh water
T2	861	300	23	Seawater
T3	1061	500	26	Seawater
T4	1124	563	28	Seawater
T5	1360	799	33	Seawater
T6	1496	935	35	Seawater

### Expression of iron metabolism genes in the head kidney of Atlantic salmon

Extraction of total RNA from the head kidney was performed as reported by Caruffo et al. [6]. Briefly, total RNA was extracted from 50 to 100 mg of homogenized head kidney tissue (Tissuelyser, Qiagen) using TRIZOL Reagent (Invitrogen) and incubated for 30 min at 37 °C with 20 units of RQ1 RNase-Free DNase (Promega) to remove residual genomic DNA. Then, the pellet was washed with 75% ethanol and carefully dried at room temperature for 5 min. The RNA was resuspended in 30 µL of nuclease-free water at 65 °C and finally stored at − 80 °C. The quality and quantity of the total RNA were determined using an Epoch spectrophotometer (Biotek, Thermo Fisher Scientific, USA) and validated by a 260/280 ratio above 1.8. Integrity was confirmed by running an aliquot of the RNA samples in a denaturing agarose gel stained with ethidium bromide and observing the ribosomal subunits. Two micrograms of total RNA was used as the template for reverse transcription reactions to synthesize single-strand cDNA using SuperScript ΙΙ (Invitrogen, Thermo Fisher Scientific, USA) according to standard procedures. The cDNA produced was diluted to 100 ng and used as the template for qPCR, with primers designed against transferrin (*Tf*), transferrin receptor (*TfR*), ferritin (*fer*) and hepcidin (*hamp*). PCRs were carried out on a CFX96 Touch Real-Time PCR Detection System (Bio-Rad) using KAPA SYBR^®^ FAST qPCR Master mix under the following conditions: 95 °C for 5 min followed by 35 cycles of 94 °C for 15 s, 60 °C for 15 s and 72 °C for 20 s. Primers for the elongation factor 1 alpha gene (EF1A) were used as the normalizer gene as previously reported by Pulgar et al. [5]. To determine the relative expression levels of iron genes, samples obtained at T1 to T4 were compared between vaccination conditions prior to the onset of SRS mortality.

To determine the bacterial load in the salmon head kidney, we used all times sampled (T1–T6) to evaluate differences in bacterial burden between vaccination conditions before and after the onset of SRS mortality, as reported by Karatas et al. [16]. At least seven biological (fish) and three technical (qPCR) replicates per condition were statistically analysed via the method described by Pfaffl et al. [17] and adapted by Talke et al. [18]. The primers utilized in this study are listed in Additional file 1.

### Transferrin gene editing by CRISPR/Cas9 in the SHK-1 cell line

SHK-1 cell line (ECACC 97111106) was obtained from the European Collection of Authenticated Cell Cultures (ECACC). Transferrin gene editing by CRISPR-Cas9 in the SHK-1 cell line was recently reported by our research group [19]. Briefly, a common guide RNA (crRNA sequence (5′ − 3′): CCTGCAGCCCATCATTGCAG) against transferrin-a (tfa) (GenBank accession LOC100136560 ssa03; 18924571–18932338) and transferrin-2 (tf2) (GenBank accession LOC106599563 ssa03; 18857195–18866421) was jointly electroporated using Cas9 RNP-based protocol in the SHK-1 cell line (1 µM RNP; 1600 V, 10 ms, and 3 pulses), as described by Gratacap et al. [20]. These mutations resulted in truncated versions of all transferrin proteins encoded in the Atlantic salmon genome (Additional file 2). The cell line simultaneously edited to tfa and tf2 was named *TF-KO* (> 80% INDEL), whereas the unmodified line was named wild-type *TF-WT*. For Sanger sequencing, genomic DNA was extracted with a Dynabeads DNA DIRECT Universal Kit (Invitrogen), and PCR was performed according to the manufacturer’s instructions (Q5^®^ Hot Start High-Fidelity 2X Master Mix (NEB)). The 3D models for the *TF-WT* isoforms and the *TF-KO* were built from the primary sequence via the AlphaFold 3 webserver [21]. Structures were aligned using FoldMason multiple structure alignment tool webserver [22] with default settings (gap opening penalty = 10, gap extension penalty = 1). The resulting alignment was visualized with ChimeraX [23].

### In vitro infection of SHK-1 cell lines (TF-KO and TF-WT) with *P. salmonis*

In vitro infection of SHK-1 cell lines (*TF-KO* and *TF-WT*) with *P. salmonis* was performed according to Caruffo et al. [6]. Briefly, *P. salmonis* LF-89 (ATTC VR-1361) was obtained from the American Type Culture Collection (ATCC) and cultivated at 18 °C in solid and/or liquid SRS broth media with constant stirring at 180 rpm. Each subculture was confirmed as *P. salmonis* by Gram staining and the RFLP assay [24]. After 4 days, the cultures were diluted to an optical density (OD_620_) of 0.05 in 5 mL of liquid media and incubated at 18 °C. The absorbance was measured with Infinite^®^ 200 PRO NanoQuant (Tecan^®^) equipment. For infection, 1 × 10^3^ SHK-1 cells were seeded on coverslips and cultured at 18 °C in Leibovitz’s L-15 medium supplemented with 5% FBS (Gibco) and 0.1 μM 2-mercaptoethanol (Sigma‒Aldrich, Germany) without antibiotics in 24-well plates (Corning). Twenty-four hours later, approximately 80% confluent cells were inoculated with stationary phase *P. salmonis* at a multiplicity of infection (MOI) of 100 (1 cell: 100 bacteria). SHK-1 cells from both groups, not inoculated with bacteria, were also cultured as infection controls (*n* = 3). After 24 h, the cells were washed twice with cold PBS and then incubated for 60 min with L-15 medium plus gentamicin (100 µg/mL) to eliminate extracellular bacteria. To evaluate the cytopathic effect (CE) caused by *P. salmonis*, SHK-1 cells were observed under an optical inverted phase contrast microscope AE31 (Motic, China) to follow the progression of the infection by image analysis (Moticam BTU10, Motic). At the median lethal time (LT₅₀), where approximately half of the wild-type SHK-1 cells died because of *P. salmonis* infection, we captured cell culture images of ten random fields for each condition, which allowed us to count *P. salmonis* containing vacuoles (PCV), a typical cytopathic effect of this infection. Cell viability was quantified using the trypan blue exclusion assay (Gibco) [1] on the basis of the principle that viable cells with intact membranes exclude trypan blue, whereas nonviable cells with compromised membranes take up the dye and appear blue under light microscopy. For quantification of the bacterial load and expression of iron metabolism genes in SHK-1 cells (for the different conditions), total RNA was obtained from SHK-1 cell monolayers and isolated via the EZNA Total RNA Kit I (OMEGA Biotek), which was used as a template for reverse transcription reactions to synthesize cDNA using All-In-One 5X RT MasterMix (Amb) according to the manufacturer’s recommendations. The qPCRs were performed according to the procedures described in the “Expression of iron metabolism genes in the head kidney of Atlantic salmon” section. Data obtained from at least three biological replicates were considered for statistical analysis.

### Transcriptomic differences between TF-KO and TF-WT

To determine the effect of transferrin editing on the abundance of transcripts associated with iron metabolism in the SHK-1 line, we performed RNA-Seq in biological duplicates, which was recently reported by our group [19]. Briefly, total RNA obtained from the *TF-KO* and *TF-WT* cell lines was sequenced on a NovaSeq 6000 (Illumina). The paired-end (150 bp) reads of the two experimental groups were evaluated using the RaNA-Seq platform [25], which mapped the transcripts to the *Salmo salar* Atlantic salmon genome (Ssal_v3.1). Normalized expression values and differential expression analysis were performed with the DESeq2 package, which was used to determine differentially expressed iron transcripts between the *TF-KO* and *TF-WT* cell lines. Iron-transcripts with an adjusted *p* value < 0.05 and absolute log_2_-fold change > 1 were considered differentially expressed between the groups. Network and enrichment analyses were carried out via STRING software [26]. Protein‒protein interactions were inferred on the basis of experimental evidence, curated databases, coexpression, and co-occurrence, applying a medium confidence threshold of 0.4. Enrichment analyses were carried out via the UniProt annotated keyword database, which includes all differentially expressed genes (DEGs) with a false discovery rate (FDR) < 0.05.

### Statistical analysis

To compare the survival curves between the SS and SIA groups, a Kaplan‒Meier or log‒rank (Mantel‒Cox) survival analysis was performed. To establish differences in the bacterial and transcriptional abundances of genes associated with iron metabolism between the SS and SIA groups, for each time point, Student’s *t* test was used on the basis of at least seven biological replicates per condition. To compare TF-KO and TF-WT cell lines exposed and not exposed to *P. salmonis*, observable cytopathic effects were analysed by two-way analysis of variance (ANOVA) followed by Bonferroni’s comparison test from 10 representative images (taken at random) from each condition. Bacterial abundance and transcript analyses were performed with at least three biological replicates per condition, and statistical significance was assessed by two-way ANOVA. Comparisons between the number of cells for each measurement time between TF-KO and TF-WT were evaluated by Student’s *t* test on the basis of at least three biological replicates and three techniques per condition. All the graphs were generated via GraphPad Prism 8.0.2 (GraphPad Software, Inc.). *P* values < 0.05 were considered statistically significant.

## Results

### Vaccination against *P. salmonis* is correlated with decreased bacterial load and increased abundance of iron metabolism transcripts

**Figure 2:**
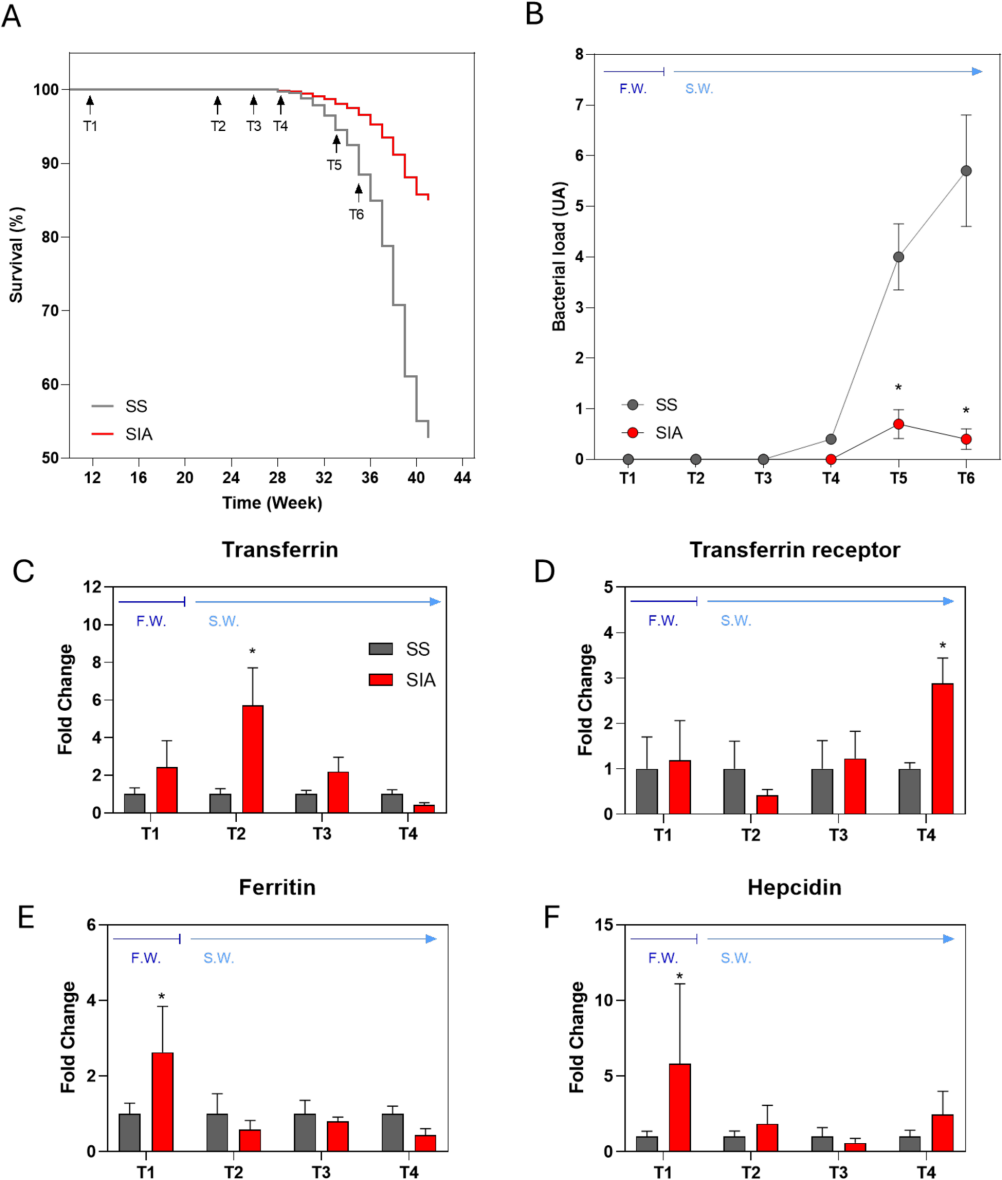
**Vaccination from SRS correlates with decreased bacterial load and increased abundance of iron metabolism transcripts*.* A** Survival percentage of fish subjected to the SS (gray lines) and SIA (red lines) vaccines. Survival was monitored on a daily basis for 44 weeks, and 6 sampling times (arrows) were established depending on the postvaccination ATUs. **B** Bacterial load (arbitrary units) of *P. salmonis* in head kidney samples from fish vaccinated with SS (grey circles) or SIA (red circles) at different sampling times. The circle represents the mean ± SD of six observations measured in at least two independent experiments. **C**–**F** Increased abundance of iron metabolism genes in the head kidney of Atlantic salmon from fish vaccinated with SS (grey bars) or SIA (red bars) at different sampling times. **C** Transferrin, **D** transferrin receptor, **E** ferritin, and **F** hepcidin mRNA abundances were evaluated, with each column representing the mean ± SD of at least seven biological replicates per condition. An asterisk indicates statistically significant differences between conditions at *p* < 0.05 (Student’s *t* test).

As shown in Figure 2A, no significant differences in survival were observed between vaccinated and unvaccinated fish up to 28 weeks after seeding (T4). However, after this time point, vaccinated fish exhibited significantly greater survival than unvaccinated fish did (85% vs. 52%, respectively) by the end of the study (week 41). To confirm that the observed mortalities were due to an SRS outbreak, we collected head kidney samples, the tissue with the highest bacterial tropism, at all study time points (T1–T6) and quantified the bacterial load by qPCR. As shown in Figure 2B *P. salmonis* was undetectable until T3, whereas at T4, the bacterium was detected at low levels in the SS group, which was consistent with the absence of mortality before T4. Notably, after T4, the bacterial load increased significantly in the SS group compared with the SIA group at T5 and T6, highlighting a correlation between the observed mortality differences and bacterial burden. On the basis of these findings, we aimed to determine whether differences in mortality and bacterial load between the vaccination groups were associated with changes in the expression of iron metabolism-related genes before the onset of mortality. As shown in Figures 2C–F, all four iron metabolism genes, *ferritin*, *transferrin receptor*, *transferrin*, and *hepcidin*, were upregulated in the SIA group relative to the SS group at least at one sampling time point. Interestingly, *ferritin* and *hepcidin* were expressed at elevated levels in freshwater (early postvaccination stage), whereas *transferrin* and its receptor were upregulated in seawater at T1 and T4, respectively, in the SIA group compared with the SS group.

### Transferrin mutant salmon phagocytes exhibit a protective phenotype against infection with *P. salmonis*

**Figure 3:**
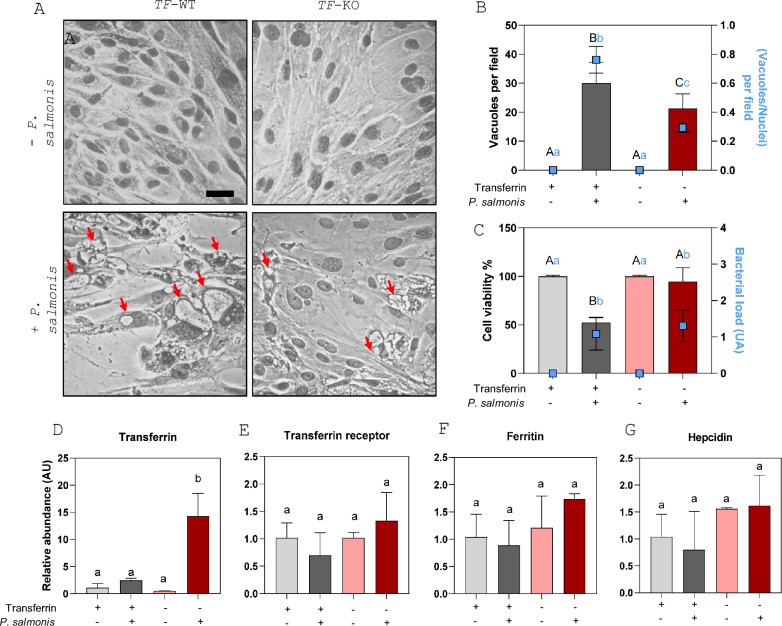
**Transferrin mutant salmonid phagocytes exhibit a protective phenotype against infection with** ***P. salmonis***. **A** Representative microphotograph of SHK‑1 *TK-*WT and *TF-KO* cells exposed and not exposed to *P. salmonis* until the median lethal time (LT₅₀) in the wild-type SHK-1 cell population; scale bar = 10 μm. The red arrows are indicative of *P. salmonis* containing vacuoles (PCV). B-G The grey columns represent the *TF-WT* cells (light grey, unexposed/dark gray, exposed to *P. salmonis*), whereas the red columns represent the *TF-KO* cells (light red, unexposed/dark red, exposed to *P. salmonis*). **B** The left axis shows the number of *P. salmonis* vacuolar cells (PCV) per field, and the right axis represents the number of *P. salmonis* vacuolar cells divided by the number of nuclei per field (blue light squares). Each column and square represent the mean ± SD of ten representative images per condition. Different letters (uppercase for the left axis and lowercase for the right axis) indicate statistically significant differences between conditions at *p* < 0.05 (two-way ANOVA). **C** The left axis shows the SHK-1 viability percentage (columns), and the right axis (blue squares) represents the relative load of *P. salmonis* (arbitrary units, AU). Each column and square represent the mean ± SD of at least three biological replicates per condition. Different letters (uppercase for the left axis and lowercase for the right axis) indicate statistically significant differences between conditions at *p* < 0.05 (two-way ANOVA). **D**–**G** Relative abundances of the iron metabolism factors transferrin, transferrin receptor, ferritin, and hepcidin, respectively. Each column represents the mean ± SD of at least three biological replicates. Different letters indicate statistically significant differences between conditions at *p* < 0.05 (two-way ANOVA).

Given that *P. salmonis* is a ferrophilic bacterium and that transferrin is a plasma iron transporter, we used CRISPR/Cas9 to generate transferrin-knockout (*TF-KO*) Atlantic salmon phagocytes. This allowed us to compare the performance of the mutant (*TF-KO*) and wild-type (*TF-WT*) cell lines during in vitro infection with *P. salmonis*. Infection progression was characterized on the basis of the cytopathic effect (CE) observed in infected cells, a phenomenon associated with the formation of *P. salmonis*-containing vacuoles (PCVs) in SHK-1 cells. The results revealed a reduction in both the number and size of PCVs in *TF-KO* cells infected with *P. salmonis* compared with those in *TF-WT* cells (Figure 3A). Quantification of PCVs per field and the vacuole-to-nucleus ratio per field revealed averages of 30.1 and 0.75 in *TF-WT* cells, whereas *TF-KO* cells presented significantly lower values of 22.2 and 0.30, respectively (*p* < 0.05) (Figure 3B). This reduction in the vacuolar cytopathic effect correlated with a significant difference in cell viability at the end of the assay. Specifically, compared with unexposed cells, *TF-WT* cells inoculated with bacteria presented a 48.3% decrease in viability (*p* < 0.001), whereas the viability of *TF-KO* cells was similar to that of unexposed cells (Figure 3C). With respect to the expression of iron metabolism-related genes, as shown in Figure 3D, none of these transcripts exhibited significant changes in response to either mutation or bacterial exposure, except for transferrin, which significantly increased in abundance in the *TF-KO* mutant line upon *P. salmonis* infection (*p* < 0.05).

### Transferrin mutants in salmon phagocytes are enriched in metal-binding-associated processes

**Figure 4:**
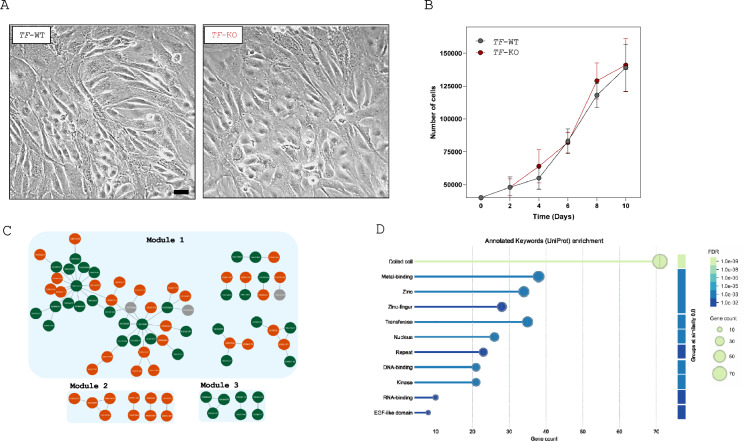
**Salmon phagocyte mutants for transferrin are enriched in metal-binding-associated processes. A**, **B** Comparison between SHK-1 *TF-KO* and *TF-WT* cells. **A** Representative photographs of cell monolayers after 10 days of growth. **B** Growth curves of SHK-1 *TF-WT* (gray circles) and *TF-KO* (red circles) cells measured every two days for 10 days. Each circle represents the mean ± SD of three biological and three technical replicates per cell line. Comparisons between conditions were performed via Student’s *t* test on the basis of at least three biological replicates and three techniques per condition and time. *P* values < 0.05 were considered statistically significant. **C** In the interaction network, the colors of the nodes represent upregulated (orange) and downregulated (green) genes among *TF*-KO and *TF*-WT, whereas the edges represent relationships between the encoded proteins on the basis of coexpression and experimental evidence (STRING). **D** Functional enrichment was performed for genes that were differentially expressed between *TF*-KO and *TF*-WT via the keywords annotated in UniProt.

As shown in Figures 4A and B, no significant differences in phenotype or growth were observed between the mutant and wild-type cell lines. However, differential gene expression analysis (RNA-seq) revealed 311 differentially expressed genes (DEGs) between *TF-KO* and *TF-WT* phagocytes, with 142 upregulated and 169 downregulated genes (Additional file 1).

These DEGs were subjected to network and functional enrichment analyses to identify gene clusters and functional processes that might explain the protective phenotype of the *TF-KO* cell line against infection. As illustrated in Figure 4C, network analysis revealed 81 nodes (genes) and 70 edges distributed across three modules. Module 1 consists of 64 interconnected genes with increased (orange) or decreased (green) expression in the *TF-KO* cell line relative to *TF-WT* (mixed response), which is distributed in four subnetworks. Module 2 is composed of two subnetworks of 10 exclusively upregulated genes, whereas module 3 is composed of 7 exclusively downregulated genes in *TF-KO* compared with *TF-WT*. Consistent with previous findings, no subnetworks were specifically associated with iron metabolism. However, within Module 1, we identified nodes related to metal regulation, including *metal-response element-binding transcription factor 2* (MTF2) and *AN1-type zinc finger protein 4-like* (ZFAND4), the latter being the most highly connected nodes in the network (degree 14). Interestingly, Module 2 comprises proteins involved in cell cycle regulation, RNA/DNA synthesis, and processing, including *deoxycytidylate deaminase-like isoform X1* (DCTD), which requires zinc for its catalytic activity. In contrast, Module 3 contains proteins associated with RNA processing and transport, such as *transcription coregulator RNA-binding protein EWS-like isoform X1* (EWSR1), whose activity also depends on zinc binding (Additional file 1). Subsequent functional enrichment analysis of the network's DEGs revealed significant enrichment for metal-binding, zinc-binding, and zinc-related processes, as shown in Figure 4D. Additionally, we observed greater nuclear genetic material binding (RNA and DNA binding) and enzyme activities related to transferases and kinases in the *TF-KO* cell line than in the *TF-WT* cell line.

## Discussion

SRS, caused by *P. salmonis*, remains a significant challenge in Chilean aquaculture. In the present study, we evaluated the impact of a combined inactivated and live attenuated SRS vaccine on fish survival, bacterial load, and iron metabolism-related gene expression during commercial salmon farming. Vaccination is one of the primary strategies for controlling infections in aquaculture, despite uncertainties regarding its long-term efficacy against intracellular pathogens [2, 27]. Nevertheless, field studies assessing the effectiveness of vaccines against SRS are scarce; hence, our initial trial was conducted under commercial production conditions. Our findings demonstrated that a pentavalent inactivated vaccine combined with a monovalent live-attenuated *P. salmonis* vaccine (SIA) significantly increased survival rates (85%) compared with those of the sham-vaccinated group (SS, 52%) during a natural SRS outbreak (Figure 2A). As this study was conducted under field conditions, where vaccination is mandatory [28], and given that we employed a commercial pentavalent vaccine routinely used in salmon farming, it was necessary to include the SS vaccine (identical in composition to the SIA vaccine but lacking *P. salmonis* antigens) as a reference. This allowed us to control a potential cross-protective immune response from SRS and to adequately control the efficacy of the antigens against *P. salmonis*. Given the multifactorial nature of fish mortality under field conditions, we subsequently sampled the head kidney at six early time points (T1–T6) before the outbreaks and quantified the *P. salmonis* bacterial load to confirm that the observed mortality was infection related. As shown in Figure 2B, the bacterial load increased in both vaccinated groups after T4, with significantly higher levels in the SIA group than in the SS group at T5 and T6. These results were consistent with the differences in mortality between the vaccination groups, and all dead fish presented a signology consistent with SRS through indirect immunofluorescence or necropsy (Additional file 3); thus, we inferred that these differences were attributable to vaccine efficacy.

Afterwards, we examined whether vaccine efficacy was correlated with changes in the transcript abundance of iron metabolism-related genes in the head kidney. Interestingly, transcripts encoding ferritin, transferrin receptor, transferrin, and hepcidin were upregulated in the SIA group compared with the SS group at least at one time point (Figures 2C–F), suggesting a link between vaccine efficacy (or bacterial burden) and the transcriptional regulation of these genes. Given that transferrin mRNA levels are significantly more abundant in postsmolts Atlantic salmon than in fish in the freshwater phase [29] and that transferrin expression is modulated in response to infections and iron availability [30, 31], we generated transferrin-knockout (*TF-KO*) Atlantic salmon phagocytes from SHK-1 cells [19] to assess their response to *P. salmonis* infection in vitro. As shown in Additional file 2, one mutant line (mutant TVIII) exhibited a truncation in exon 3 of both paralogues encoding Atlantic salmon transferrins (Additional files 2A and B). Structural alignments of the wild-type (*TF-WT*) and mutant (*TF-KO*) proteins revealed that the mutation disrupted both iron-binding sites (Additional file 2C), suggesting that the *TF-KO* phagocytes were incapable of transporting or loading iron into transferrin. This mutant was selected because both paralogous genes were disrupted, preventing potential phenotypic complementation, a critical consideration for gene function studies in polyploid organisms such as salmonids [32].

To assess the response of *TF-KO* compared with that of *TF-WT* phagocytes following *P. salmonis* infection, we performed an in vitro infection assay and monitored the cytopathic effects (CEs) in both cell lines. These CEs, which are well characterized in *P. salmonis* infections of the SHK-1 cell line, include an increase in the size and number of *Piscirickettsia*-containing vacuoles (PCVs), ultimately leading to phagocyte death [1, 6, 33]. As depicted in Figures 3A–C, compared with TF-WT cells, *TF-KO* cells presented reduced PCV formation and increased cell viability. Despite these differences in cytopathic effects and cell viability, the bacterial burden remained unchanged between *TF-KO* and *TF-WT* cells, indicating that transferrin depletion resulted in an infection-tolerant phenotype independent of the availability of intracellular iron transferrin-binding for the pathogen. Additionally, the transcript levels of transferrin, transferrin receptor, ferritin, and hepcidin did not significantly differ between *TF-KO* and *TF-WT* cells (Figures 3D–G), suggesting that cellular iron homeostasis was not markedly affected by transferrin disruption. These findings suggest that the absence of transferrin reduces cytopathic effects, limits infection progression, and prevents *P. salmonis*-induced death in SHK-1 cells without altering the expression of key iron metabolism-related genes. This effect may be attributed to the fact that iron uptake in phagocytes is not solely dependent on transferrin; alternative pathways, such as divalent metal transporter 1 (DMT1)-mediated uptake of nontransferrin-bound iron (NTBI), may compensate for the loss of transferrin [34].

Given that transferrin is involved not only in iron transport but also in processes such as inflammation and cell signalling [35, 36], we conducted a comparative analysis of morphology, growth rates, and transcriptional profiles (RNA-Seq) between *TF-KO* and *TF-WT* cells to better contextualize our findings. Since transferrin has been shown to influence cell proliferation [37, 38], we first assessed whether its absence affects phagocyte growth dynamics, which could contribute to the observed differences in tolerance to *P. salmonis* infection. However, as shown in Figures 4A and B, no significant differences in cell morphology or proliferation rates were observed between *TF-KO* and *TF-WT* cells, indicating that transferrin-dependent pathways do not influence this phenotype in SHK-1 cells. From the point of view of transcriptional regulation, we identified 311 DEGs between TF-KO and TF-WT cells, of which 81 DEGs were connected in three expression modules (Figure 4C). Consistent with previous observations, no subnetworks were specifically linked to iron metabolism, and several nodes related to general metal regulation, including metal response element-binding transcription factor 2 (MTF2), were detected. Notably, MTF2 function and regulation depend on zinc and copper availability [39, 40] and have been implicated in various biological processes [41]. In agreement with these findings and the well-established interactions among transition metals [42, 43], functional analysis revealed significant enrichment for metal-binding, zinc-binding, and zinc-related pathways (Figure 4D). On the other hand, we observed differential enrichment of RNA/DNA binding functions, transferase and kinase activities, and EGF-like domains in *TF-KO* cells compared with those in *TF-WT* cells. Although these functions are broad and involve numerous biological pathways, several proteins associated with them have been implicated in mechanisms of intracellular pathogen infection. For example, the host protein EWSR1, which was downregulated in our study, is hijacked by SARS-CoV-2 to facilitate viral replication [44]. It has also been identified as a CRE site-binding protein for hepatitis C virus (HCV), and its silencing has been shown to inhibit viral replication and the production of infectious particles [45]. Although evidence in fish is limited, EWSR1 has been reported to be downregulated in the brains of groupers (*Epinephelus malabaricus*) during persistent/tolerant infection with nervous necrosis virus (NNV) [46], suggesting that downregulation of this gene might play a role in host tolerance to infection with intracellular pathogens.

Finally, the most upregulated gene in *TF-KO* cells was MVP, which encodes the major vault protein. This protein is the main component of a highly conserved ribonucleoprotein complex found in most eukaryotic organisms. This protein is associated with the resistance of human lung epithelial cells to infection with *Pseudomonas aeruginosa* [47], and Atlantic salmon is highly expressed in the skin mucosa of fish affected by amoebic gill disease (AGD) [48]. Although the function of these proteins has not been clearly established for salmon, their changes in expression levels in *TF-KO* phagocytes suggest that these molecular adaptations may contribute to the observed protection against *P. salmonis* infection, aspects that need to be functionally tested in the future.

While the precise role of transferrin in modulating the tolerance of salmon phagocytes to *P. salmonis* infection remains complex, the findings of this study lay a foundation for incorporating iron metabolism—particularly transferrin-dependent pathways—into the rational design of vaccines targeting this pathogen. By enhancing our understanding of host–pathogen interactions, these insights may inform the development of more effective and sustainable strategies for controlling SRS, ultimately contributing to the long-term health and productivity of salmon aquaculture.

## Supplementary Information

**Additional file 1.** **Primer sequences, DEG list, coexpression modules, and enrichment analysis.** The table contains the list of primers used in this study, the list of genes differentially expressed between *TF*-KO and *TF*-WT, the data on the interactions between these genes (and their expression module assignments) and the data from the enrichment analyses.

**Additional file 2.** **Transferrin gene, protein, and structure alignment in TF-KO and TF-WT. A** Sequencing results of the genes encoding transferrin in Atlantic salmon (*TF*-KO and *TF*-WT). **B** Alignment of the protein sequence encoded by *TF*-KO and *TF*-WT. **C** Alignment of the protein structure encoded by *TF*-KO and *TF*-WT.

**Additional file 3.** **Immunofluorescence and clinical findings in SRS-affected *Atlantic salmon* in seawater. A** Indirect immunofluorescence assay (IFAT) of brain and liver tissues from eleven deceased fish at T6. The IFAT results are presented by tissue and detection intensity and are classified as follows: +  = 1–10 bacteria per field; +  +  = 11–50 bacteria per field; and +  +  +  =  > 50 bacteria per field. **B** Representative images of the necropsy-induced mortalities. The image above shows skin lesions in the fish associated with SRS. The bottom left image shows anterior and posterior renomegaly along with pale hepatic nodules. The bottom right image displays a pale liver with whitish nodular formations, visceral fat congestion, and splenomegaly. All 11 fish presented clinical signs consistent with salmonid rickettsial septicaemia (SRS). Assays were conducted by ETECMA (Health Reports No. 19-20399 RPGT-09-01 and CCS-JS-201-069-1).

## Data Availability

All information and supplementary information have been detailed and accessible. For any further inquiries, the authors are willing to provide them.
