# Supplementary figures and images for: Transferrin knockout reveals a tolerance phenotype against Piscirickettsia salmonis in Atlantic salmon phagocytes
Source: Vet Res. 2025 Sep 25;56:180. doi: 10.1186/s13567-025-01607-8 (PMC12465926; doi:10.1186/s13567-025-01607-8)

## Slide 1
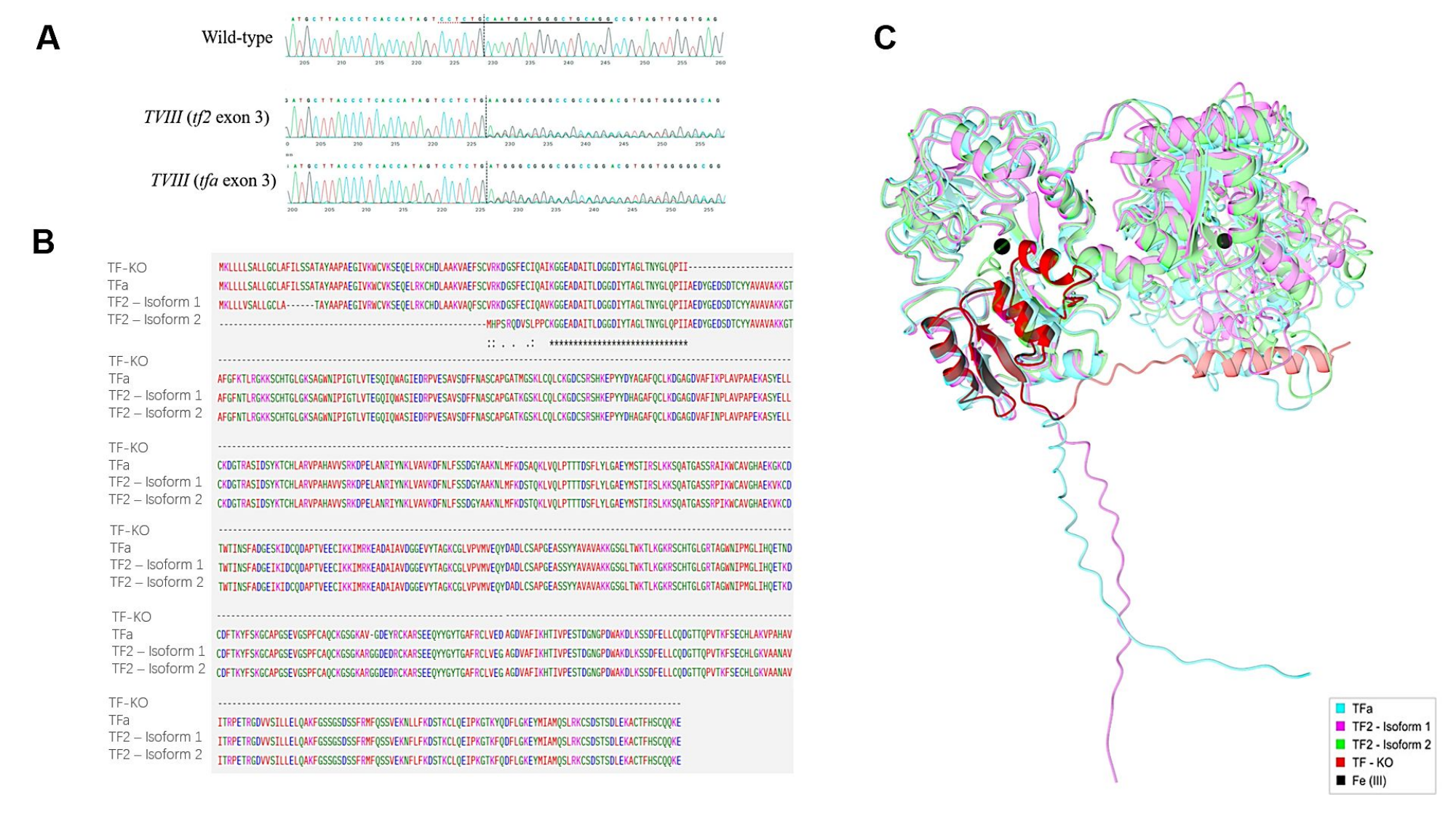

Supplement: Supplementary file 2 — Additional file 2. Transferrin gene, protein, and structure alignment in TF-KO and TF-WT. A Sequencing results of the genes encoding transferrin in Atlantic salmon (TF-KO and TF-WT). B Alignment of the protein sequence encoded by TF-KO and TF-WT. C Alignment of the protein structure encoded by TF-KO and TF-WT. [file 13567_2025_1607_MOESM2_ESM.pptx]

## Slide 1
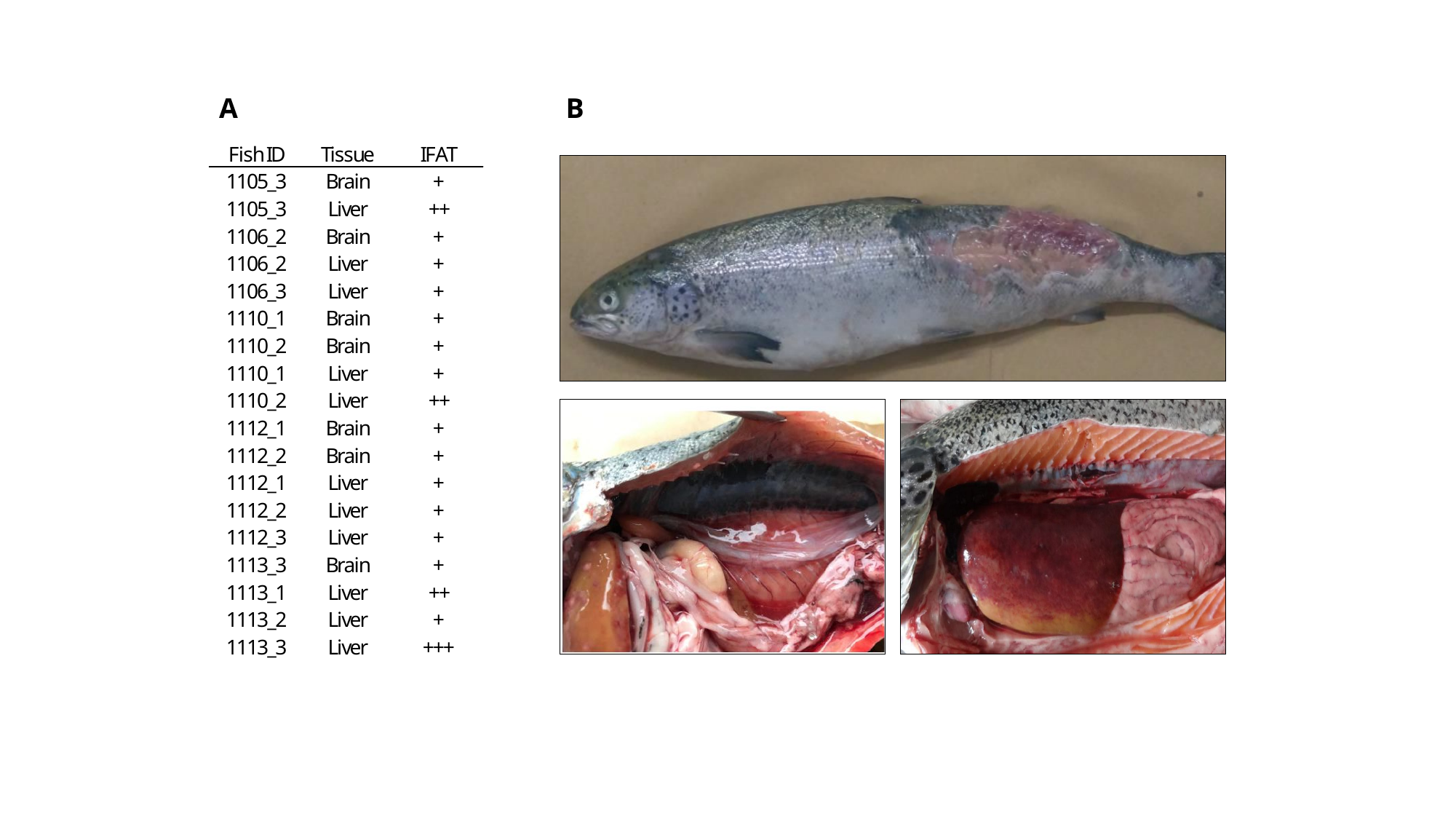

A
B

Supplement: Supplementary file 3 — Additional file 3. Immunofluorescence and clinical findings in SRS-affected Atlantic salmon in seawater. A Indirect immunofluorescence assay (IFAT) of brain and liver tissues from eleven deceased fish at T6. The IFAT results are presented by tissue and detection intensity and are classified as follows: + = 1–10 bacteria per field; + + = 11–50 bacteria per field; and + + + = > 50 bacteria per field. B Representative images of the necropsy-induced mortalities. The image above shows skin lesions in the fish associated with SRS. The bottom left image shows anterior and posterior renomegaly along with pale hepatic nodules. The bottom right image displays a pale liver with whitish nodular formations, visceral fat congestion, and splenomegaly. All 11 fish presented clinical signs consistent with salmonid rickettsial septicaemia (SRS). Assays were conducted by ETECMA (Health Reports No. 19-20399 RPGT-09-01 and CCS-JS-201-069-1). [file 13567_2025_1607_MOESM3_ESM.pptx]
